# Copy number variants in kiwifruit *ETHYLENE RESPONSE FACTOR/APETALA2 (ERF/AP2)*-like genes show divergence in fruit ripening associated cold and ethylene responses in *C-REPEAT/DRE BINDING FACTOR*-like genes

**DOI:** 10.1371/journal.pone.0216120

**Published:** 2019-05-13

**Authors:** Kularajathevan Gunaseelan, Peter A. McAtee, Simona Nardozza, Paul Pidakala, Ruiling Wang, Karine David, Jeremy Burdon, Robert J. Schaffer

**Affiliations:** 1 The New Zealand Institute for Plant and Food Research Ltd, Auckland, New Zealand; 2 School of Biological Sciences, University of Auckland, Auckland, New Zealand; 3 The New Zealand Institute for Plant and Food Research Ltd, Motueka, New Zealand; Institute of Crop Science, CHINA

## Abstract

The *ETYHLENE RESPONSE FACTOR/APETALA2* (*ERF/AP2*) transcription factors have been shown to control a wide range of developmental and environmental responses in plants. These include hormonal responses to ethylene and Abscisic Acid (ABA) as well as to cold and drought. In *Actinidia chinensis* (kiwifruit), ripening is unusual: although it is sometimes classed as a climacteric fruit (ethylene-associated ripening), much of fruit ripening occurs independently from autocatalytic ethylene production. Initiation of ripening appears to be strongly developmentally controlled and modulated by low temperature. In this study, fruit treated with different temperatures showed an increase in soluble sugar accumulation, and a corresponding increase in *ß-AMYLASE* (*BAM*) genes (predominantly *BAM3*.*2* and *BAM9*) with lower temperatures. To investigate the potential role of the *AP2/ERF* gene family in the control of fruit ripening in kiwifruit this family was investigated further. Using the new genome annotation and further genome sequence analysis we identified 226 *ERF*-like genes, 10 *AP2L*/*RAV*-like genes and 32 *AP2*-like genes. An RNA-seq screen from kiwifruit of different maturities, and following treatment with ethylene and temperatures between 0 and 16°C, revealed 4%, 26% and 18% of the *ERF*-like genes were upregulated by maturation, ethylene and cold temperatures, respectively. Focusing on the *C-REPEAT/DRE BINDING FACTOR* (*CBF*) cold master regulators, nine potential genes were identified based on sequence similarity. Five of these *CBF*-like genes were found in a copy number variant (CNV) cluster of six genes on chromosome 14. Expression analysis showed that two homeologous genes (*ERF41* and *ERF180*) increased in abundance with cold and ethylene, while the cluster of CNV *CBF*-like genes had lost the ability to respond to cold and increased only with ethylene, suggesting an evolutionary progression of function of these genes.

## Introduction

Kiwifruit berry ripening is unusual; although it is sometimes classified as a climacteric fruit (ethylene associated ripening), much of the fruit ripening occurs independently from autocatalytic ethylene production [1]. As the fruit develops on the vine it progresses from maturation into the first phase of ripening (Phase 1) independently of autocatalytic ethylene and involves a progression of starch breakdown, colour change, and loss of firmness [1]. Once almost fully soft there is an autocatalytic ethylene phase (Phase 2) associated with further softening and the production of aroma volatiles [1, 2]. The progression of Phase 1 ripening can be accelerated by a cold treatment [3–6]. On the vine, Phase 1 ripening may be influenced by both fruit development and the environment. After the fruit reaches physiological maturity, if the fruit is exposed to ethylene, Phase 1 ripening is accelerated [2, 7] and, depending on the stage of development and the amount of ethylene, the fruit can be pushed into Phase 2 ripening.

The molecular mechanisms underpinning fruit ripening are still the focus of much research. It has been long established that ethylene is one of the key ripening hormones in many fruits, and many downstream genes controlling each of the ripening characters, such as colour, texture, starch metabolism and flavour volatiles, have been extensively reported [8]. The ethylene pathway has been well characterised in *Arabidopsis* in which it was observed that one of the key genes that control the transcriptional response to ethylene is an AP2 domain containing the ERF class of genes [9]. Combining genetic knowledge with genome sequences has allowed all the ERFs to be mined. In many plant species it has been shown that the *ERF/AP2* gene family is large; in *Arabidopsis* there are 122 members [10]; in rice, there are 139 [10]; in tomato, there are 146 *ERF*/*AP2/DREB*-like genes [11, 12] and 77 *ERF*-like genes [11, 13]. Based on phylogenetic alignment of the DNA binding domain, the *ERF*-like genes have been classified into ten subgroups in rice and *Arabidopsis* [10] and this classification has since been applied to other species. In tomato, *ERF*-like genes were subdivided into nine clades (A-J) [11, 13]. Extensive *ERF*-like gene functional analysis has revealed diverse roles in environmental, hormonal and plant developmental activities [10, 13]. Some *ERF*-like genes play a role in tomato fruit ripening [13–15]. There have been a few studies of *ERF*-like genes in kiwifruit: initial mining of EST libraries identified 14 ripening related *ERF*-like genes [16], while the use of the draft kiwifruit genome gene model [17] identified a total of 119 *ERF*-like genes [18].

Whilst the ethylene pathway has been very well described in plants [19, 20], the mechanism by which plants measure temperature is still being resolved; however, it is known that plants have adaptive mechanisms that allow them to survive at different temperatures. In many plant species, cold temperatures (below 0°C) are lethal, but some plants are able to survive when they are exposed to a cold (e.g. 4°C) period of acclimation prior to a freezing event. The mechanism by which plants protect themselves from freezing temperatures is well characterised [21], and is achieved through the expression of the *ERF*-like *DREB1*-like class of transcription factors named *C-REPEAT/DRE BINDING FACTOR* (*CBF*) [22]. The *CBF* gene induction can occur in plants within 15 minutes of exposure to low temperature (4°C) [23]. Once induced by cold temperatures, the expression of *CBF*s tends to decrease relatively slowly over the following days [24]. Over-expression of the *CBF1* or *CBF3-like* genes is sufficient to induce constitutive cold acclimation, resulting in plants that can survive when placed directly in freezing temperatures with no acclimation [25, 26]. There has been one *CBF-like* gene described in kiwifruit, *AcCBF1*, that was unusually shown to increase in expression only after 20 days of cold treatment [27]. Less is known about how plants respond to chill (8–10°C) temperatures at the molecular level.

Based on a kiwifruit genome manual annotation project, it was found that 90% of the original gene models [17] were inaccurate [28], suggesting that previous studies in gene families may need to be updated. In this study we aimed to understand more fully how developmentally controlled fruit ripening is influenced by ethylene and temperature. Using the newly annotated kiwifruit gene models [28] and further genome searches, this study will focus on *ERF/AP2*-like genes and starch breakdown (*BAM*). Using new and existing RNA-seq data from maturing fruit, and postharvest fruit that have been treated with ethylene or different temperatures, we aim to identify candidate genes that have changes in RNA abundance associated with these treatments.

## Materials and methods

### Identification of *ERF-like* and *β-AMYLASE* (*BAM*) genes in the kiwifruit genome

The new gene models of *A*. *chinensis* var. *chinensis* genotype Red5 [28] were compared with individual *AP2*-like and *ERF*-like genes from each subgroup [10] using BLASTP [29]. Gene lists from these multiple searches were then condensed into a list of potential *AP2/ERF*-like genes. A further search of the translated Red5 gene model genomic sequence using a protein BLAT search was also conducted with divergent AP2 DNA binding domain sequences to identify any further genes that were missed during the manual annotation process. Missing genes were manually annotated using the WebApollo software [30], and a new accession number was assigned. Finally the list of published *ERF*-like genes [18] was checked to make sure that none of the genes was missing.

The predicted peptide sequences were generated from the gene mining, and these were aligned using MUSCLE alignment in Geneious 10.0.3 (www.geneious.com). Genes without a full AP2 DNA binding domain were removed from the gene lists. The 68-amino acid DNA binding domain was extracted for each gene and a phylogenetic relationship was built using PHYML [31] using the JTT substitution model and default settings. This alignment was repeated with the published ERF protein sequences [18] from the kiwifruit genome resource (http://bioinfo.bti.cornell.edu/cgi-bin/kiwi) and the NCBI database (https://www.ncbi.nlm.nih.gov). Gene names were then transferred to the new gene models. These alignments identified the AP2-like and ERF-like groups described by Nakano et al. [10]. These separate subclades were extracted, realigned and optimum alignments obtained from an output of 1,000 bootstrap datasets. Comparison of peptide sequences in the CBF group from *Arabidopsis* (CBF1 AT4G25490, CBF2 AT4G25470, CBF3 AT4G25480) [21], grape vvCBF1 (AAW58104.1) VvCBF2 (AIL00572.1), VvCBF4 (AIL00786.1) [32], tomato SlCBF1 (Solyc03g026280.3), SlCBF3 (Solyc03g026270.3), SlDREB1A (Solyc03g124110.2) [33] and potato StCBF1 (ABI74671.1) StCBF4 (ACB45083.1) [34] and apple MdCBF1 (ART85558.1), MdCBF3 (ARO50175.1), MdCBF4 (ART85561.1) were used in the phylogeny.

β-AMYLASE (BAM) predicted proteins were mined by homology to the *Arabidopsis* protein sequences from the Red5 kiwifruit genome by the BLAT function of the WebApollo software [30]. Results were compared with previously published sequences [35]. A peptide Geneious Alignment was generated for the kiwifruit *BAM* gene models and the *Arabidopsis BAM* genes in Geneious 8.1.2 (www.geneious.com). The phylogenetic analysis was performed as described above for *ERF*-like genes, and the *Bacillus cereus* BAM protein (P36924) was used to root the tree [36].

### Fruit treatments

All fruit used were *Actinidia chinensis* v. *chinensis* ‘Hort16A’ defect free. RNA-seq data from maturing and ethylene treated fruit presented in [7] were remapped to the new gene models [37]. A new trial investigating temperature effects was conducted.

The fruit maturation RNAseq samples were from fruit harvested off the vine on the following days; Maturity 1–147 Days After Full Bloom (DAFB), 2–168 DAFB, 3–175 DAFB, 4–224 DAFB and covered time points when the fruit were transitioning between non-ethylene responsive and ethylene-responsive maturity.

The ethylene treated fruit were harvested when the fruit were fully responsive to ethylene, but not producing endogenous ethylene (231 DAFB). All fruit were kept at 21°C. Fruit were harvested (0d) and treated for 1 day with 100ppm ethylene (1d) then moved into an ethylene free environment and sampled 1 day later (2d) and 2 d later (4d). For the temperature treatments, fruit were harvested when at a soluble solids content (SSC) of 6.5° Brix during mid-April (2011) from an orchard under commercial management at the Plant & Food Research orchard, Te Puke, New Zealand. Immediately after harvest, 10 fruit were assessed for flesh firmness and SSC as described in [38]. Fruit were randomised into 28 batches of 20 fruit and placed into plastic pocket packs (plixes) in single layer fibreboard trays and placed into seven different temperature controlled cabinets set at 0, 3, 8, 10, 12, 14 and 16°C (four packs per temperature). Twenty random fruit were removed 24 h, 48 h and 192 h (8 days) after placement at different temperatures, separated into two biological replicates of 10 fruit, and quarter tissue slices (containing skin, outer pericarp inner pericarp, seed and core) were cut from the middle of each fruit, chopped and immediately snap frozen in liquid nitrogen for later expression analysis. At the 192-h (8-day) sample, the final batch of 20 fruit was assessed for SSC and flesh firmness as described in [38].

### RNA isolation and mRNA sequencing

Total RNA was isolated from the frozen fruit samples using a method to extract RNA from pine tree needles [39]. RNA quality and quantity were analysed using Agilent 2100 Bioanalyzer (Agilent Technologies, Santa Clara, CA, USA). Ten micrograms of DNase-treated (TURBO DNA-free kit, Ambion) total RNA was sent to Macrogen (South Korea) for cDNA library preparation, barcoding and sequencing on a single lane of Illumina HiSeq2000, yielding 200 bp RNA-Seq reads. For each of the RNA-seq sets, a minimum of 10M reads were obtained (S1 Table) RNA-seq data were cleaned according to the method of McAtee et al. [7] and aligned to the new gene models [28] using STAR v0.8.1. TPM (Transcripts Per Kilobase Million) values were calculated by first dividing the mapping count for each gene within each library by the length of the same gene (in kilobases) to generate a Read Per Kilobase (RPK) value. The sum of the RPK values within each library was then calculated and divided by a million to achieve a ‘per million scaling factor’. The RPK values within each library were then divided by their respective ‘per million scaling factor’ to yield the final TPM values. Differential expression analysis was performed in R (version 3.2.4) using the DESeq2 package [40].

Primers to validate the RNA-seq results were designed to unique regions of genes identified as changing in the RNA-seq screen (S2 Table), and standard curves were calculated for each primer set. Quantitative PCR (qPCR) was performed as described by Atkinson et al. [2] Kiwifruit *ACTIN* (*Acc08082*.*1*, PSS26667.1) (S2 Table) was used as a reference gene [41]. RNA from fruit at harvest was used as a calibrator.

## Results

### Identification of *ERF/AP2*-like genes in kiwifruit

Mining the newly annotated kiwifruit gene models [28] for *ERF/AP2*-like genes identified a total of 256 gene models. When the translated genome was searched using divergent AP2 domain protein sequences, a further 13 putative genes were found. Eleven of these 13 putative genes appeared to be full length, so were given new Acc model numbers (S1 File); two (*AcERF149* and *AcERF164*) were not full length and ended at a genome construction join, so were assumed to be real models that were limited by genome assembly quality. Both these fragments had a full AP2 DNA binding domain and were therefore included in the following analysis. Initial phylogenetic alignment of the DNA binding domain protein sequence separated them into three main clades comprising 226 *ERF-like* genes, 5 *RAP-like* genes, 32 *AP2-like* genes, and 5 *PLETHORA-like* genes (S3 Table).

**Fig 1:**
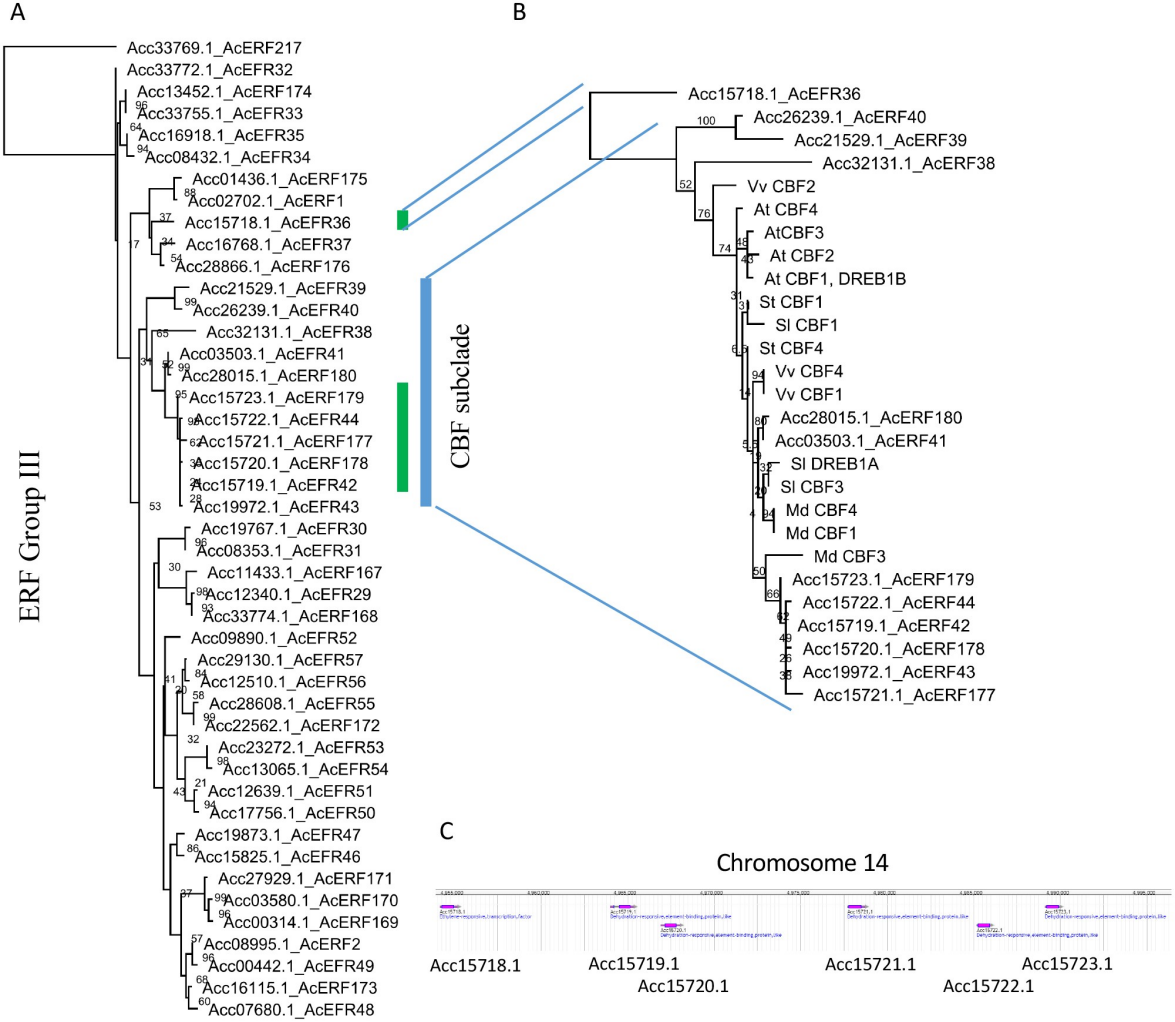
**A**. **Phylogenetic alignment of the DNA binding domain of Group III *ERF* genes**. The ancestral gene Acc33769.1 was used as an outgroup. Bootstraps of 1000 iterations given. B. A subset of Group III representing *C-REPEAT/DRE BINDING FACTOR*-like genes aligned to the known CBF-like predicted DNA binding domains from *Arabidopsis* (CBF1 AT4G25490, CBF2 AT4G25470, CBF3 AT4G25480), grape vvCBF1 (AAW58104.1) VvCBF2 (AIL00572.1), VvCBF4 (AIL00786.1), tomato SlCBF1 (Solyc03g026280.3), SlCBF3 (Solyc03g026270.3), SlDREB1A (Solyc03g124110.2) and potato StCBF1 (ABI74671.1) StCBF4 (ACB45083.1), and apple MdCBF1 (ART85558.1), MdCBF3 (ARO50175.1), MdCBF4 (ART85561.1), were used in the phylogeny, AcERF36 (Acc157718.1) as an outgroup. The blue vertical line represents the CBF-like clade, and green lines represent the Copy Number Variation event. C. Gene duplication of a CBF-like gene was identified on Chr14.

The 226 *ERF-like* genes were aligned to the *119 ERF* published *ERF* genes, and the new gene models were named with the existing *ERF* nomenclature. From this original set it appeared that the previously published genes (*AdERF11* and *AcERF108*) and (*AcERF44* and *AcERF45*) were represented by single gene models *Acc03601*.*1* and *Acc15722*.*1* respectively. *Acc03601*.*1* is likely to have been given two models because of sequence differences in the two species from which they were initially cloned: *AdERF11* was identified from an EST from *A*. *chinensis* var. *deliciosa* ‘Hayward’ and *ERF108* was from the *A*. *chinensis* var. *chinensis* ‘Hongyang’ gene model. *Acc15722*.*1* was part of a cluster of copy number variants and it will be presented later. The initial phylogenetic analysis confirmed the 10 EFR groups described in *Arabidopsis* and rice [10], but compared with *Arabidopsis* and rice, there has been a large clade expansion in kiwifruit especially in the Group III, V, VII, VIII, IX, X subclades (Table 1). The new gene models were named sequentially from *ERF120* to *ERF239*. From the initial phylogenetic alignment the subclades were extracted, realigned with the representatives of the subgroups A-J (S3 Table), and bootstrap tests of 1,000 iterations were conducted to generate a robust alignment for each group. In each of these, the ancestral gene Acc33769.1_AcERF217 was used to root the tree (Group III is shown in Fig 1A, all the rest are in S1 Fig).

**Table 1:** Numbers of *AP2/ERF* genes by cluster group and species.

ERF group	Published kiwifruit	Kiwifruit this study	*Arabidopsis*	Rice
I	4	11	10	9
II	10	15	15	15
III	30	44	23	26
IV	5	10	9	6
V	10	21	5	8
VI	10	11	12	9
VII	7	10	5	15
VIII	13	29	15	13
IX	23	49	17	18
X	5	26	11	13
**Total ERF**	**117**	**226**	**122**	**132**
other				7
RAV		5		
PTH		5		
AP2		32		

In addition to the in-depth phylogenetic analysis, each sequence was analysed for the repressive EAR domains previously identified in *ERF* protein sequences. The EAR domain comprises (L/F) DLN (L/F)x P primary structure [42] and are typically found at the C terminal end. Twenty-two genes in two ERF groups (Group II and Group VIII) were identified as having the EAR repressive domain (Group II—seven out of 15 and Group VIII 16 out of 29) (S3 Table).

### Gene duplications within the ERF/AP2 gene families

When examining the clusters (Fig 1, S1 Fig) most genes were aligned in homeologous pairs, consistent with the genome duplication event described in the Red5 genome [28]. There were a number of additional loci that appeared to be locally duplicated (identified by sequential gene model numbering), consistent with a Copy Number Variant event (CNV). Some of these duplications appear to be recent, with the duplicates appearing closely aligned in the phylogenetic tree (S1 Fig), whilst others appear to be older with greater sequence divergence, and were no longer clustering so closely together. One noticeable CNV was in a gene cluster that most closely resembles the *CBF* genes from other species [22] (Fig 1B).

### Assessment of fruit with different temperature treatments and identification of *ß-AMYLASE* genes

**Fig 2:**
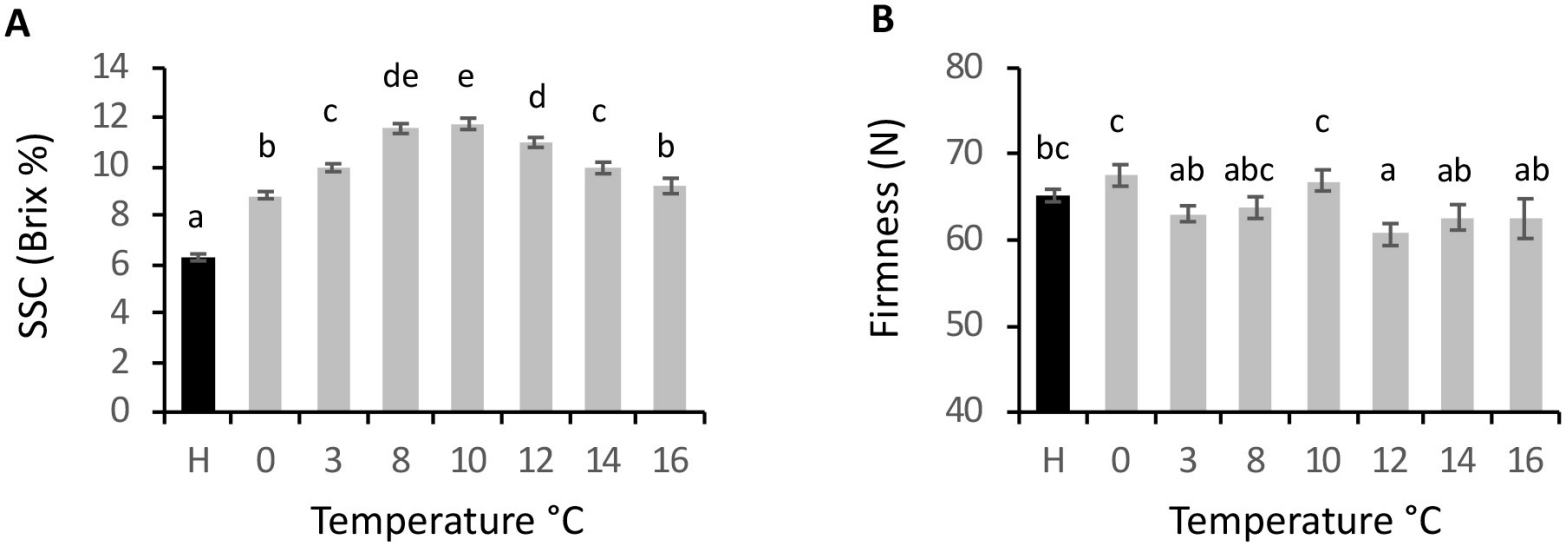
Fruit physiology. A. Soluble solids content (SSC) and B. firmness of *Actinidia chinensis* var. *chinensis* ‘Hort16A’ kiwifruit at harvest (AH) and after 8 days at temperatures between 0 and 16°C. n = 20, ± s.e.m. letters represent significance p < 0.05.

To identify which genes may be involved in the fruit ripening process, RNA-seq screens were examined. Firstly, previously published yellow-fleshed kiwifruit RNA-seq data from maturing and ethylene-treated fruit samples of ‘Hort16A’ [7] were re-analysed using the new gene models and normalised to TPM. Secondly, as no equivalent ‘Hort16A’ RNA-seq data were available for kiwifruit treated with different temperatures, tissue was harvested for an RNA-seq screen from physiologically mature fruit (harvest sample) and following a 2-day temperature treatment at seven temperatures between 0°C and 16°C. In all these fruit samples, following the 2-day holding period, there was no significant difference in ripening associated SSC or fruit firmness from the harvest samples. To confirm that the fruit behaved similarly to those described in the literature (ripening more quickly with cooler temperatures [3, 4]), a further post-harvest measurement was taken following eight days of temperature treatment. Eight days after harvest, it was found that SSC had increased to between 8.8 °Brix and 12 °Brix. Fruit that had been stored at 10°C reached a maximum SSC of 12.0 °Brix, while those that were treated at 16°C and 0°C were at 9.1 °Brix and 8.8 °Brix, respectively. This indicated that at all temperatures the breakdown of starch had progressed, with starch breakdown progressing faster in temperatures between 8 and 12°C (Fig 2A). During this short holding period there was no significant change in the fruit firmness (Fig 2B).

**Fig 3:**
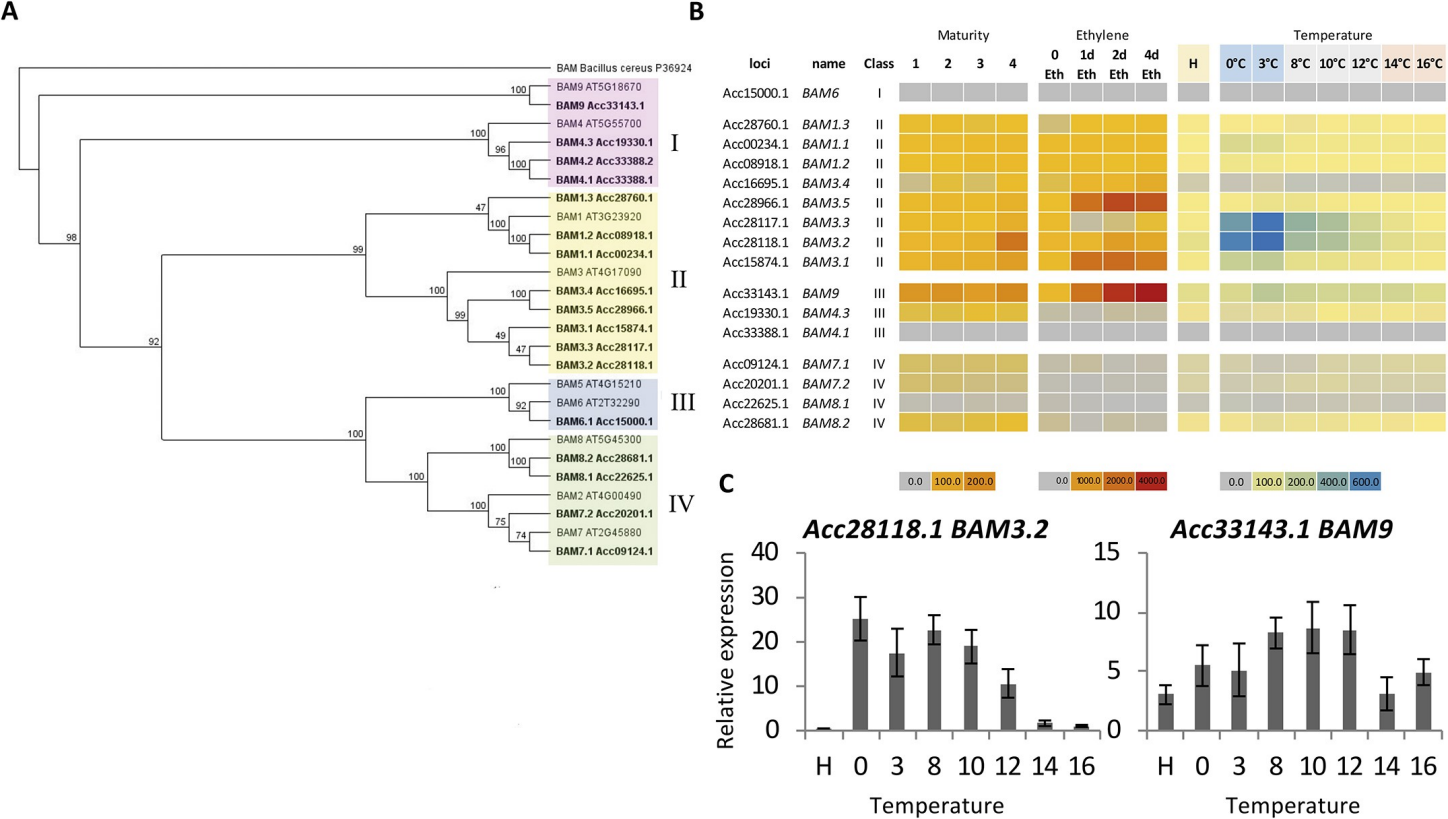
Classes of *ß-AMYLASE* genes. A. Phylogenetic alignment of BAM predicted proteins showing the four main subclades of genes. B. Expression analysis of RNA-seq reads of BAM genes from *Actinidia chinensis* var. *chinensis* ‘Hort16A’ kiwifruit of different maturities as defined in [5], and following ethylene (100ppm) and temperature treatments. C. qPCR verification of gene expression of BAM3.2 and BAM9 at different temperatures, n = 4, ± SEM.

As there was a clear starch degradation pattern, the regulation of starch breakdown by the β*-AMYLASE* (*BAM*) class of genes was investigated. Using the new gene models generated in the recently improved kiwifruit genome [28], sixteen *BAM*-like gene models associated with 15 loci (one predicted model has an alternately spliced isoform) were identified. Phylogenetic analysis separated these into the four different subclasses of *BAM* genes, in agreement with *Arabidopsis* [36]. One clustered in the Group I class (*BAM6*), eight were in the Group II class (three *BAM1* and five *BAM3*), four were contained within the Group III class (two *BAM4* (plus one alternate spliced variant) and one *BAM9*), and four in the Group IV class (two *BAM7* and two *BAM8*). Each gene was named according to the literature when possible [35, 36] (Fig 3A). A tandem duplication of the class II *BAM3* genes was observed (*BAM3*.*2* and *BAM3*.*3*). A point to note is that there have been multiple *BAM3*—like genes reported with different names. For example, *BAM3*.*5* has also been named *AcßAMY1; BAM3*.*3* named *AcßAMY2* and *BAM3L*; and *BAM3*.*4* named *BAM3* in recent publications [43, 44]. Hopefully this new naming will address these discrepancies.

The RNA-seq screen suggested that most of the *BAM*-like genes were expressed, with only two having no reads associated with maturity, ethylene treatment or temperature. Four *BAM*-like genes had very low RNA abundance (<1 TPM), three had moderate abundance (>1 TPM and <10), while seven had high abundance (>10 TPM) (Fig 3B). All the high abundance *BAM* genes were class II and class III *BAM* genes. The class II and class III genes were upregulated by low temperature, while the class IV genes were not. In the class II genes, the tandemly duplicated *BAM3*-like genes (*BAM3*.*2* and *BAM3*.*3*) were the most abundant. Two other *BAM3* genes were highly expressed (*BAM3*.*1* and *BAM3*.*5*) and two *BAM1*-like genes were highly abundant (*BAM1*.*1* and *BAM1*.*2*). Of the class III genes, *BAM9* had the highest transcripts, with the *BAM4* genes having low to moderate expression (Fig 3B). To verify the RNA-seq screen, qPCR confirmed expression of *BAM3*.*2* and *BAM9* genes (Fig 3C).

### Transcriptional regulation of *ERF/AP2*-like genes in fruit of different maturity, and following ethylene and temperature treatments

The expression patterns of all the *ERF/AP2* like genes were examined in an RNA-seq screen from the maturity trial, the ethylene-treated trial [7] as well as the RNA-seq screen from the different temperatures. The RNA-seq screen identified that in these fruit samples 21% of the genes (56) had no reads at any sampling point. Thirty-five percent of the genes (94) had very low expression (a TPM of less than 1 at any given time point), 22% of the genes (59) had a moderate expression in at least one time point (>1 TPM and <10 TPM), and 20% of the genes (55) had an expression greater than 10.

To identify those genes that showed variation in abundance with maturity, with ethylene or temperature, the magnitude of difference was measured. If there was a change of greater than two-fold but less than four-fold and a maximum TPM of <1, it was described as a weak activation/repression; more than four-fold change to a maximum TPM of <10 was described as a medium activation/repression; and a strong activation/repression was >16-fold change with a maximum TPM >10. In total, 22 genes showed a change as the fruit matured, whilst 76 (22%) showed a change with ethylene treatment, 49 (15%) responded to a cold 0–3°C treatment, and 15 responded to a chill 6–12°C treatment (S4 Table, Table 2). The genes in the ethylene treatment and the cold treatment were found to have the highest numbers of medium and strong activation or repression.

**Table 2:** Summary of expression of *AP2/ERF* genes.

	Change with maturity	Change with ethylene	Change with cold	Change with chill
+	4	17	8	3
++	3	25	16	6
+++	2	16	16	0
**Sum +**	**9**	**58**	**40**	**9**
-	7	6	5	6
--	5	10	3	0
---	1	2	1	0
**Sum -**	**13**	**18**	**9**	**6**

**Fig 4:**
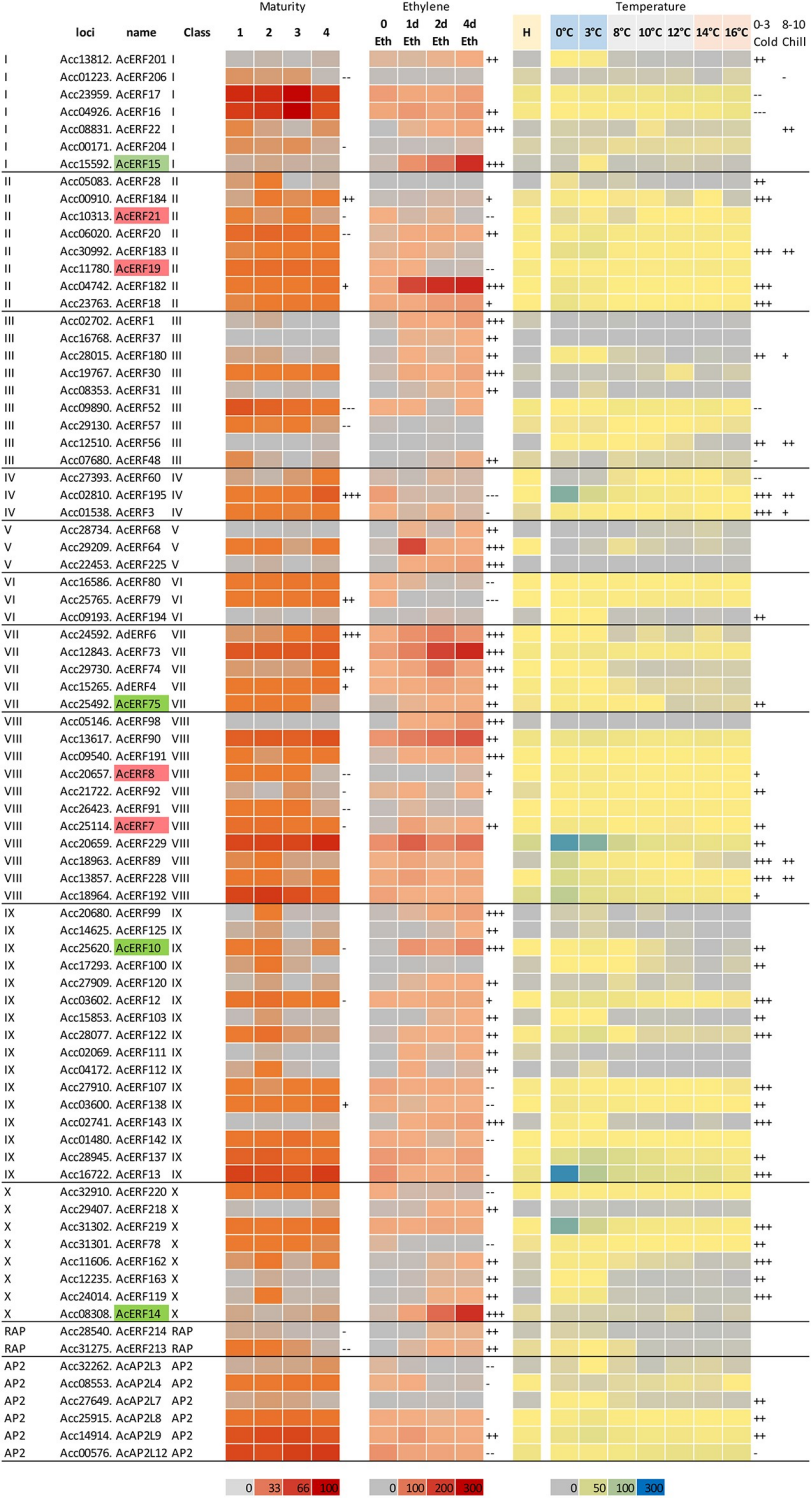
RNA-seq expression analysis. 81 ERF/AP2-like genes that show a medium or strong change in expression during *Actinidia chinensis* var. *chinensis* ‘Hort16A’ fruit maturation and following ethylene (100ppm) and temperature treatments. + indicates the genes are activated, and—that the genes are repressed. Gene names in red represent genes previously suggested to be repressors of ripening, while gene names in green represent proposed activators.

Genes showing a medium and strong activation are shown in Fig 4. It is noteworthy that each of the groups are represented by at least one gene that is activated by cold, and at least one gene activated by ethylene. These selected genes demonstrated a combination of cold-only regulation, ethylene-only regulation or ethylene and cold regulation. Here we focused on the CBF class of genes found in Group III. The CBF class was chosen because functional analysis has shown that in model species these are master regulators of the cold response [22], and in this study they appear to be part of a copy number duplication event; these often allow the evolutionary change in function.

**Fig 5:**
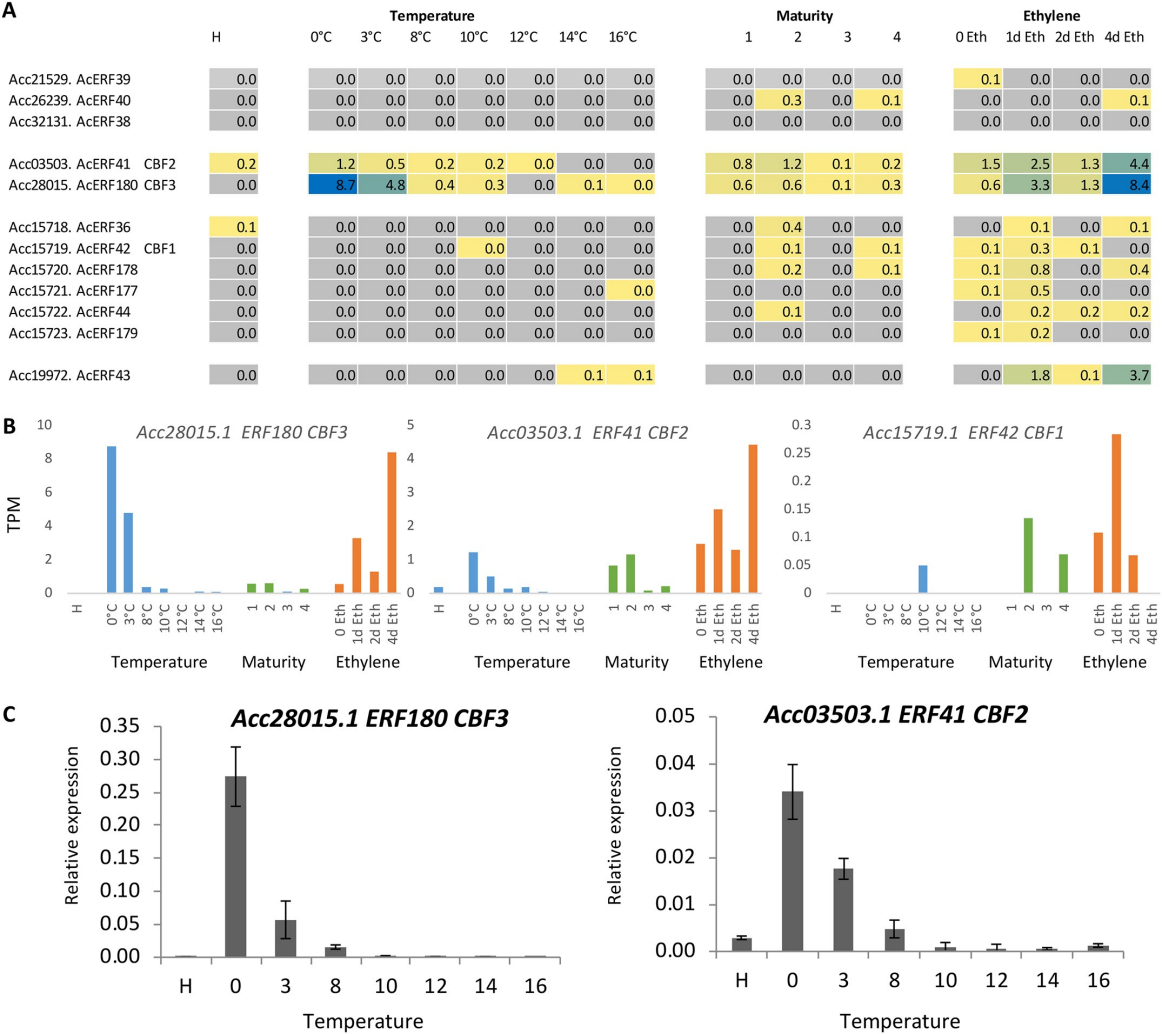
A. RNA-seq expression analysis of *CBF*-like genes in *Actinidia chinensis* var. *chinensis* ‘Hort16A’ kiwifruit, during fruit maturation and after ethylene (100ppm) and temperature treatments. B. Graphs of gene expression of two candidate *CBF*-like genes and the published *CBF1* (*ERF42*), showing the cold and ethylene control of expression. C. qPCR verification of the two cold-induced *CBF* genes, n = 4; ± SEM.

Of the CBF, cluster two (*Acc03503*.*1_ERF41* and *Acc28015*.*1_ERF180*) seemed to be acting in a manner similar to *CBF*-like genes, having very low abundance at harvest, and responding rapidly to a cold treatment of 0° or 3°C (Fig 5A and 5B). The expression of these two genes was validated with qPCR (Fig 5C). It is noteworthy that the previously published *CBF1* gene [27] (*Acc15719*.*1_EFR42*) was not expressed in cold-treated fruit, having no mapping sequence reads in these fruit. Indeed none of the genes in the duplicated set was expressed with the cold treatment, suggesting that the function of these genes has evolved from the cold response.

When the expression of the *CBF* cluster was examined in the fruit-maturation and ethylene-treated samples, the *CBF*-like genes were activated by ethylene, with the ethylene response at a similar magnitude to the cold response with *Acc28015*.*1*. The ethylene response was less than the cold response in *Acc03503*.*1*, and the ethylene response was lower but still measureable in the *Acc15719-23* cluster. This suggests that the cold response has been lost in these duplicated genes.

## Discussion

During kiwifruit ripening there is an interesting dynamic of temperature and speed of ripening. Traditional post-harvest practices utilise this dynamic, using cold temperatures to preserve fruit, through slowed fruit metabolism and reducing rots. While cold storage is very successful for fruit storage, it is becoming clear that the cold as a treatment in itself can have a profound influence on kiwifruit ripening beyond simply slowing metabolism [3, 4]. This suggests that while not apparent at low temperatures around 0°C because of the slowing of metabolism, faster ripening occurs at intermediate chill-temperatures of 8–12°C. It has been shown that fruit ripening in kiwifruit is controlled by developmental, hormonal and environmental cues. Two dominant cues are ethylene and temperature, with both ethylene and a cold and chill treatment promoting ripening. By examining the *ERF/AP2* gene family in kiwifruit we have found that across the *ERF/AP2* gene family there are a considerable number of genes that are regulated at the transcriptional level by ethylene and temperature. In total, 81 genes representing each ERF group changed during fruit ripening and or cold/chill treatment, suggesting that key regulators are included in this gene family. Previous work reported in the literature has identified and named just over half the ERF genes [18] and consistent with previously demonstrated expression analysis, there was a consistency in the expression profiles observed here. Zhang *et al*. [18] proposed that in group I *AcERF15*, was a potential ripening activator, strongly induced by ethylene (Fig 4); in Group II the EAR domain containing *AcERF19* and *AcERF21* demonstrated a decrease in expression with ethylene, consistent with being potential ripening inhibitors; Group VII *AcERF75* was observed to be ethylene and cold induced; Group VIII *AcERF7* and *AcERF8* were shown to be ethylene and cold induced; Group IX *AcERF10* was also reported to be a potential ripening activator, was also shown to increase with ethylene and cold; the strongly activated Group X *AcERF14* was also postulated to be a potential ripening activator *ERF6* has also been shown to be upregulated in mature fruit by ethylene in ‘Sanuki Gold’, ‘Hayward’ and ‘Rainbow Red’ cultivars [43, 44].

In tomato, ERFs belonging to the subgroups *SlERF*.*E* and *SlERF*.*F* (E class) were shown to be regulated by ethylene and some (E1, E2 and E4, and F2) appeared to be modulated by *RIN* and *NOR* [13]; the E class are represented by Cluster group VII and F class by Cluster group VIII. In this study four and 11 genes in this class were selected as ethylene-responsive genes, with the best aligned genes *SlERF*.*E1* aligning to *AcERF4* and *AcERF75*, both of which show an increase with ethylene, suggesting possible orthologues. In the F Class (Group VIII) F2 showed the closest homology to *AcERF9* [16] and a CNV cluster of three genes *AcERF185*, *AcERF186*, *AcERF187*. *ERF9* was moderately expressed and weakly activated by ethylene (<2 fold, S4 Table). While the closely related *AcERF187* was expressed also, it did not change during these conditions and the other two genes (*AcERF185* and *AcERF186*) were not expressed in ripening fruit. In subgroup *SlERF*.*H*, *SlERF*.*H1* (*LeERF1*) [15] also has an ethylene and ripening effect. The H class represents Group V, of which the closely related gene, *AcERF225*, appears to be strongly ethylene regulated.

Studies by [45] showed increased activity of ß-AMYLASE in response to cold. Also another study related CBF-mediated cold response and sugar dynamics [46]. This suggests that there may be a close link between the increased levels of BAM and cold in other plant species. It has been shown previously that cooler temperatures can promote fruit ripening [4, 47, 48]. The expression patterns of the *BAM* genes suggest that there is a very strong upregulation of *BAM3*.*2* and *BAM3*.*3* with cooler temperatures, with a maximum expression of *BAM3*.*2* at between 0 and 8°C (Fig 3C). This is consistent with previous reports of expression in other kiwifruit cultivars showing that *BAM3*.*3* (*AcBAMY-2*) increased with cooler temperatures [44]. The expression of the *BAM9* gene was lower than the *BAM3-like* genes but had a maximum expression at 8–12°C. The ripening physiology suggests that the 8–12°C temperature range is the most effective promoter of ripening, suggesting that either the *BAM9* has a dominant starch degradation effect or that the lower 0–3°C temperatures inhibit the enzymic starch breakdown and that the 8–12°C range is point of high expression and warm enough for the enzymes to be highly active.

At the molecular level little is known about how plants respond to chill (8–10°C) temperatures; however, much more is known about the control of expression at the 0–3°C temperatures. This cold temperature response has been shown to be part of the CBF class of genes. One may speculate that the cold induction of genes at the 0–3°C range is controlled by the *ERF41* and *ERF180* CBF-like genes (renamed here as *CBF2* and *CBF3*), given the close sequence similarity, and expression analysis. The gene duplication of the larger *CBF-like* gene family appears to have allowed the evolutionary divergence of expression of this group, moving from the strongly cold-regulated *ERF180*, that unusually appears to be controlled by ethylene as well, to the less strongly cold-activated *ERF42* (and *ERFs 36*, *44*, *177*, *178*, *179*) that have maintained their ethylene activation, but do not appear to be cold regulated at all. A point to observe is that in the previous study, the reported activation of the *ERF42* (previously named *CBF1*) coincided with an increase in ethylene 20 days after storage [27], suggesting that the early cold activation report may actually have been a response to ethylene rather than cold.

Previous studies of cold stress have tightly linked CBF genes to being involved in relieving the stresses [23], often these are not single copy genes. It is shown that CNV in this gene set is not uncommon, with *Arabidopsis* having three, two of which activate the cold response and a third that represses it. In tomato, the *SlCBF* gene that activated cold in *Arabidopsis* inhibited the response in tomato, suggesting a complex mechanism of regulation of this gene in the tomato system; this appears to be mirrored by cold intolerance in tomatoes. In apple, the *CBF* genes [49] were shown to regulate the expression of the *PG1* gene, independently but additively with ethylene [48]. This is the first study linking ethylene-related ripening and the CBF response suggesting that in kiwifruit some of the ethylene and cold effects could be occurring through the same pathway.

## Supporting information

S1 Table Number of RNA-seq reads mapped to the new gene models. (XLSX)

S2 Table Primers for qPCR. (XLSX)

S3 Table Existing and new *ERF/AP2* genes in kiwifruit, highlighting those with EAR domains and duplicated genes. (XLSX)

S4 Table Expression analysis of all the ERFs. (XLSX)

S1 File New gene models for *ERF/AP2*-like genes mined from the genome. (DOCX)

S1 Fig Clusters of all the ERF gene sets. (PPTX)
