# Supplementary material for: Copy number variants in kiwifruit ETHYLENE RESPONSE FACTOR/APETALA2 (ERF/AP2)-like genes show divergence in fruit ripening associated cold and ethylene responses in C-REPEAT/DRE BINDING FACTOR-like genes
Source: PLoS One. 2019 May 13;14(5):e0216120. doi: 10.1371/journal.pone.0216120 (PMC6513069; doi:10.1371/journal.pone.0216120)
Supplement: S1 File — (DOCX) [file pone.0216120.s005.docx]

**cDNA sequence - New ERF like genes**

> Acc33766.1 AcERF208

ATGGATTTGATCAACAGCTCAAATAAAGTCAACTCGCCATCATCATCATCATCCACGTCGT

CATCGTCAAAGAGCAAAAGGAAGAATCAACAACAAAGCCAAAAAGATTTAAACAACTCAGG

GGGACTAAGGTTTATTGGGGTAAGGAGAAGGCCATGGGGCAGATACGCAGCAGAGATAAGG

GATCCTTTGACCAAAGAGAGGCACTGGCTTGGCACTTTTGACACTGCTGAGGAGGCAGCAC

TCGCGTACGATAGAGCTGCTCGCTCAATGCACATGCGCATGCAAATGGGGGACTACAATTC

CAATTCCATGCGCACTACTCGCACCAACTTCGTCTACTCTGACATGCCTCCCGGTTCCTCC

CTCACTTCCATCATCTCCCCCGATGATGAATATTCCCAACATCCTCTCCTCTTCCTAATGA

ATTCCCAGCAAAACCAACCACCACCGCCACCACCGCCCAGTGGGTTTTTTCCAGTTCCTGG

AGATGAGTGGATTCAGCGCAATACACTGACTGATCATCACAATCAGCAGCAGCAGCACCAA

CAACAGCTTTGTGATGAGCAGCAGATTATTACAGACGGTTTCATTATGCATGGAGATGTTG

GTGGTGGTGGGTCGACCAAGACCAACCAACACTACTATTGTAATGTTGATGAGGTGGAGGC

GGAGCTTCCACCATTGCCACCTGATATTTCCAATAGCTATGATTCAAGGAGCATGTGTGGG

GCCAATGTTCATGGTTCTATGTGGAACGAGGAGGACCCCATGAGTAATCTACTAATTGATA

ATGGTAGGTTTTCGGATCAGCAGACAACCACCGGGTACTACTATCATGAGGCAGAGACTGG

GAAGAGTACTGTGGGGATTGAGTCATACAATAATGTTAATGTGGGATTCTTAATGGAGTCT

GCGAGCAGCAGTGAGTTGATGAACCCGCTCTTCAGAATGACACAATAA

> Acc33767.1 AcERF216

ATGATTGAGGATGTCAAAACGCATGTGATTGCTGAGGGTTCTGTATCGAGCAGCCCCTGCC

AACCATCGAATGCGGCAATGCCAGCAAAATTCAAAGGGGTTGTGCCACAGCCAAACGGGCA

CTGGGGAGCGCAAATCTATGCAAATCACCGAAGAATTTGGCTGGGGACCTTCAAATCTGAG

ATTGAAGCTGCTATGGTCTACGACAGTGCATCGCTCAAGCTCCGGACAGGAGACAACTACA

GAAACTTTCCGTGGACCAATGTAACTGTTCACGAGCCGAAATTTCAAAGCCTTCATAGTAC

AGAAGCAATTTTGAACATGATCAGGGATGGATCGTATACATCCAAATTTGCAGATTTTCTT

GGGAGCCAAAAAGGGGAACAAGAATTAAGTGTCAACCTAATCACGGTGCATGGCCATGGAG

GATTTTCTTTTAAACTTCTTTTTCAAAAGGAATTAACCCCAAGCGATGTAGGTAAGCTTAA

TAGACTTGTCGTCCCAAAGAAATACGCAGTTAAGTACTTCCCTGTGGTGCCTGAGAGTACC

GAAGGAAATACAGATGATGGGAAGGTGGATGAGGTGCATCTCTTTTTCTACGACAGAATGA

TGAGGTTGTGGAAATTTCGGTACTGCTACTGGAAGAGCAGCCAGAGTTTTGTCTTCACTAG

GGGTTGGAATCGGTTTGTAAAGGAAAAGGGATTGAAGGAAAACGACATTATTGCTTTCTAC

GTGTGTGAGGGTCGAGAAAGAGCAAAAGAAGGCCACTCGTTTTGCCTTATCGATGTAGTTT

ACCCTGAGGGATCAACAAATTCTAGGGTGGATGAGGGGACAAGCCAGAATGTTGATATGCA

GGTGGATCTACGTCTGAAGTTGGGACAAAATCTCAATTGTTGGTTAGTTAAGAAAGATGAC

GAAGGATTTGAGAATTCAAAGGTTTTTACCAGATTGAAGCCAACTCTTAATGGGAAAAAAA

GTGTTAGGCTTTTTGGTGTTGAGCTCAATTGA

> Acc33768.1 AcERF144

ATGTACGAGCAAACAGTCTCGAACACCGATTTCGCTCTCCTCGAATCGATTCGGCGACATC

TCCTTGAGGATTCGGACATCTCAGATTTTTTTTCGGCGACCGACGCACCCAATACGCTGCC

AATTTACGACCCAAGCCGAAGCTTTGAATCAATAGTGGAGGAGCGCGTGGGGGAACGCAGC

GATCACTCTCCGCCGGTAGAGTGGCGGAAGTACAGAGGCGTGAGGCGGAGGCCGTGGGGGA

AGTTCGCGGCAGAGATTAGGGATCCGAAGAAAAGGGGAGCCAGGATATGGCTCGGGACGTA

CGAGACGTCCGAGGACGCTGCAGTCGCCTACGACCGAGCCGCGTTCAAGATACGTGGCTCC

CGTGCCAAACTTAACTTTCCGCACCTGATCGGCTCAAATAAATTTGAGCCGATCAGGGTGA

CCCCGAAGCGGAAGAAAAGCGCAGCTGGCTGCAGCTTGTCTCCAGCGGCTCCGGCCGAGTT

GGATTGTGACACATTGATAGGGGTGTTTGACTCTGAAATGGACACTGATTTTTATTGCTTG

TAA

> Acc33769.1 AcERF217

ATGGAAGAAGAACCGTCAAGTATGATTGAGGATGTCAAAACGCGTGTGATTACTGAGGATT

CTGTATCGAGCAGCCCCTGCCAACCATCGAATGCAGGGATGCATCCAATTAAAGACAACAA

AGCAATGCCAACAAAATTCAAAGGGGTTGTGCCACAGCCAAACGGGCACTGGGGAGCGCAA

ATCTATGCAAATCACCAAAGAATTTGGCTGGGGACCTTCAAATCTGAGATTGAAGCTGCTA

TGGTCTACGACAGTGCATCGCTCAAGCTCCGGACAGGAGACAACTACAGAAACTTTCCGTG

GACCAATGTAACTGTTCACGAGCCGAAATTTCAAAGCCTTCATAGTACAGAAGCAATTTTG

AACATGATCAAGGATGGATCGTATCCGTCCAAATTTGCAGTTTTTCTTGGGAGCCAAAAAG

GGGAACAAGATTTAGGTGTCAACCTAATCAAGGGGCATGGCCATGGAGGATTTTCTTGTAA

ACTTCTTTTTCAAAAGGAATTAACCCCGAGCGATGTGGGTAAGCTTAATAGACTTGTCATC

CCAAAGAAATACGCAGTTAAGTACTTCCCTGAGGTTCCTGAGAGTACCGAAGAAAATATAG

ATGATGGGAAGGTGGATGAGGTGTATCTCTTTTTCTACGACAGAATAATGAGGTTGTGGAA

ATTTCGGTACTGCTACTGGAAGAGCAGCCAGAGTTTCGTCTTCACTAGGGGTTGGAATAGG

TTTGTAAAGGAAAAGGGATTGAAGGAAAACGACATTATTGCTTTCTACGTGTGTGAGGGTC

GAGAAAGAGCAAAAGAAGGCCAGCCTTTTTGCGCTATTGATGTAGTTTATACTGGGGGATC

AAGAAATTCTAGGGTGGATGAGGGGAGAAGCCAGAATGTTGATACGCAGGTGGATCTACAT

CTGAGGTTGGGACAAAATCTCAGTTGTTGGTTAGTTAAGAAAGATGACGAAGAATTTGAGA

ATTCAAAGTTTTTTACCGGATTGAAGCCAACTCCTAATGGGAAAAAAAGTGTTAGGCTTTT

TGGTGTTGAGCTCAATTGA

> Acc33770.1 ERF95 ATGGAGAATCTTCCCCAGCTTCTTAGCAGAAACACTAGGAGAAGAAGCTCCAGATACTCCA

CTACATACCTCGGAGTTCGACGGCGGCCGTGGGGCCGGTACGCGGCCGAGATCCGAAACCC

ATTAACCAAAGAGAGACACTGGCTGGGTACATTTGACACTGCAGAGGAAGCTGCACTGGCT

TATGATCTCTCTTCCATCTCTTTTAGTGGGATTGAAAGAGCGAAAACTAATTTTCACTACC

CATTTTTGGCTCTTCCCTCCCCCTCACCGCCACCTCCGCCGCCCACGCCGTCGACTCCGGA

TTTAGAGTTGGAAGGGGAGTTGGGATGTGTTGAGGATGATGAGTCTCTTGTTATTGCCTCC

ATCTTGCAGAGCTTTGCCCTGCAGCCTAGCAACTTCTCTTTTTCATCTTGA

>Acc33771.1 AcERF109

ATGGAAGGAGGGAAGAAGAAGGCACAAGAGAATAGTGGGAGAGGAGGGGAGGCCAAGTACC

GGGGCATCCGGCGGCGGCCGTGGGGCAAGTACGCGGCGGAGATATGCGACCCATCGAAGAA

CAAGTCGCGGCTCTGGCTGGGCACGTTTGAGACGGCCGAGGAGGCAGCGCGCGCCTACGAC

CGAGCCGCGTATTCATTGAGGGGACGCCAAGCCATTCTCAACTTCCCCAATGAGTTCCATT

GCTCGAGCCCTAGCCATGGGTTAGACGGACCCTCTTGTTCTAGGGTAGGGGTTGAGTCGAG

CCAAGAGGAGCAAGTGATTGAGTTTGAGTATTTGGATGACAAGTTGTTGGAGGAGCTTCTT

GGGACCAATGAGGATCGGCATGCCAACAAAATATCAAAGTTTCCATAA

>Acc33772.1 AcERF32

ATGGATTACTCAAGTGGAGGTGGTGGTCCTAGTGGCCGCGGAGGCAATGTGGCGGCGGGGG

ATGCTCGGGGCTCAGGTAGGCACCCGGTGTACCGGGGGGTGAGACGGAGGAACAGCGGGAA

GTGGGTCTCGGAGATCCGGGAACCACGGTCGCCCAACCGGATATGGCTCGGGACCTTCCCA

ACGCCAGAGATGGCGGCGGTGGCCTATGATGTGGCAGCGCTGGCCCTGAGGGGCCGGGACA

CCGAGCTGAACTTCCCCAACTCAGCCTCCTCGTTGCCCGTGCCGATGTCCACCGCTCCTCG

CGATATCCAGGAAGCGGCGGCCAGCGCGGCTGCAGCTGCCGGGGCAGCGACAGATGCCTTG

GCTGGGTGGACTCAGGTCCACATGAATGAGAATGCGAATCGAAGTGGTGGTGGTAGTGAGT

TTGTTGATGAAGATTTGATATTTGATATGCCCAATGTTCTGGTGAATATGGCTGAAGGGAT

GCTTCTTAGCCCTCCAAGACTTAATGTTGCTGGTGATCATGACACTGCAGCTGAATATGCA

GGAGACCACAATTTGTGGAAATTCCCATAA

>Acc33773.1 AcERF210

ATGGAAGAAGCACTAAGAATGCTAAACGACAGCCTGATCCACCAGCCGGAGCCCGACCCAT

TTCTCCAACCCCTAAAGCGCGTCACCACCGCTGCCAACAAGCGCTCCCTACGTGACGGCGG

CGAAGGAGGATGCGGTACCCAGCTGAGGTACCGCGGCGTCCGGCGCCGCCCGTGGGGTCGC

TACGCCGCCGAGATTCGGGACCCGCAGTCGAAGGAGCGTCGGTGGCTGGGGACCTTCGACA

CGGCGGAGGAGGCGGCCTGCGCCTACGACTGCGCCGCCCGAGCCATGCGCGGCGTGAAAGC

CCGCACCAATTTCGCTTACCCCACCGTCACGACACTACCCCCGCCACCGTCGCTCACCGAC

AATTTCCTTTCTCGGTTTCACTGCAAGAAGCCATCTCAGACGTCTGTCAGAGACATTCCCG

CACCCACCCGTAGCTTTGTCTCCTCTCTCTCTAGCCCCCACGGTAACTTCCCTATGCCGAC

GCCGCAGAGAAGCACTTCTCTCAACACCCTTCTCTTGTCGAATCTTTTCAAGCCTTCGCTC

ACATGCGAACAATTGCCTAATGTTTCTCCCTCTACAAGTGTTTGTGGTAATGCCTATGAGA

ATTATCCTGGTTCAAATCAGATCCGACCCGAGACTGAGTATCACAATCCCGGTGAGTCTGA

CGACGGGTTAGATTTCTTCCGTTCAGAGCCGTCAGGTTCGGGGCTTTTGCAGGAGGTGCTA

AACGGGTTCTACCCGAAGCCCGCTTCGAAGGGTGACGAATTATCGGCAACCCAAAATCAGA

ATTGCACCAATGAGGCTTTTAATGAGATGATGGGAATTGAAAGCGACCATTTCGGACTGCA

CTTCGACGGGTTTGGATCGCCGGCGGTGGCTCATTCTAGCATTTCGGTGCCGGAGGAGTCA

ATGTTGTGTGAACACGTGTTTCAGTTTCACGAACTTGTTAGCGCTCGTCCTTTTCAGTT

>Acc33774.1 AcERF168

ATGGAAGGGTACTCAAGTAACAATTATGTTGGTGATGGTGGTACTGGTGGTGGTGGTGCCA

GGTCGATATACAGGGGAGTAAGGAAAAGAAAATGGGGGAAATGGGTGTCGGAGATACGCGA

GCCAGGCAAGAAAACCCGGATATGGCTAGGAAGTTTCGAGACGCCTGAGATGGCTGCCGCA

GCATATGATGCGGCCGCATTGCACCTCAGGGGTCACGGGGCGCGACTAAACTTTCCAGAGC

TGGCTCACAGTCTTCCTCAACCAGGGGGCCCGAGCGCTGAGGATGTGCGCCGGGCTGCTCA

AGAGGCCGCTTTGCGCTTCCAAAATCCCGCCTCTAGCTTTGAGGCGGGTGGCTCTAGTTCA

AATCTTGCTCCGGTCACCATAGGACTCTCCCCGAGTCAAATCCAGGCCATTAACGAATCCC

CATTGGAATCACCCAAGATGTGGATGGACCCGACTGGAGCGCTAATGCTAGAAGAGCGCGC

CTTTTTTTCTAATGATGTTGAGATGGATGAGTGGGATGAAATCACTGATGATTCCCTTTGG

GACCCATAA

>Acc33775.1 AcERF148

ATGCAAAGGCACAGCCCGCCCAAGAGACCAGAACACGGTGGAGCTTCCACGTCCCGCAACC

CCCCGTCGCGGCCTTCGCGCTTCACCCGCGAGGAGGAGACCTCCGTCATGGTCGCAGCCCT

CTCTTCCGTCATTTCCGGTTCCACGTCCCACAACCCCCCGCCACGGCCTTCGCGCTTCACC

AGCGAGGAGGAGACCTGCGTCATGGTCGCTACCCTCTCGTCCGTCATTTCCGGCTCCACGT

CAACGCAGGCGGCGGACCTGCATTTACTCATTATTCCGGAACCCGACAGGTGTCAGTTCTG

CAAATGCGAAGATTGTGTGGGATGCAACTTTTTCCCGCCAAACCCAGACGAGAAGTATGAT

AATAACAAGAACGCTGGTATTAGCAGTAATAGTAAGAATGATAATAAAAAAGGCGGGAAGA

GGAAGAAGAAGAACAAGTTTAGGGGAGTCAGGCAGAGGCCTTGGGGGAAGTGGGCGGCGGA

GATATGGAATCCGAAGCGGTCGGCACGAGTTTGGCTCGGGACCTTCGAGACGGAGGAGGAG

GCTGCCCGTGCCTATGACCGGGCCGCCATTGAATTTTGCGGGACCCGTGCCAAGCTCAATT

TCCCGGTCCCCGACAGCTCGGCGCTGGCCCAGGAGGGCCAGAACTCGGAACTGCGGGTAGA

GAGAGAAAATCCGTCGATCACGGATATGGAAATAGGAGTCATTCGGGAAGAAGATACTGAG

ACTGAGTGGATTAACACGATGATGAGCTTCGACGGGGATTGTTCGGGTTCTGCTGCCACTG

GAAATTCACACAGTTTCTAA

**New *AP2* like genes**

> Acc31415.1 AcAP2L24 ATGGAGATGATAACTTTGGTGAAGTCTGAAGTGAGCCCAGTGAGGCGCCGGTTGTGTGAAA

TGGAAGGAGACCAAAAGCAGCCACAGCAACTACTAGTTGATAAGTCCAGTGCTACTAATAC

TACTGTCAAGAGAAGCTCAAGGTTTCGTGGTGTCAGCCGATGGACTGGAAGATATGAGGCT

CACCTGTGGGACAAAGGGTCCTGGAATGTAATAACACAGAGGAAGAAGGGGAAACAAGGAG

CTTTTGATGAGGAAGAATCTGCAGCAAGAGCATACGATTTAGCTGCAATCAACCACCAGGT

TACTGATTATGAGAAAAAGATTGAGATGATGCGGAATGTAACAAAAGAGGAGTACTTGACC

TCTTTGAGACGAAGCAGTGGTTTCTCAAGAGGTGTATCCAAATACAGAGGAGCTGCAAGGC

ACCATCACAATGGAAGATGGGAAGCCAGGATAGGGAGGGTGTTTGGAAACAAGTATCTCTA

CCTTGGTACTTACAGTACCCAAGAGGAGGCTGCGTGTGCTTACGACATTGCAGCGATTGAG

TACAGAGGGATTAATGCAGACACAAGACTAAACATGGAGTCTCAGCCGGTAAATACCTTGT

CTGACCAGATTTCGAGTCAAGAACCCGAGTTCACCTTTCGCTCAAGTCCTTATACTATGGG

CAATTATGGAAACAATCCTCGGAAACAGGAAGTTCTGGAGACAAAGATAACGATTAGTCCT

TGTAATAGGTCGCCTTCCCCGACTGCGCTCGGTCTTCTCCTTAGGTCTAACATGTTTAGAG

AATTGGTGGAGAAGAATTCGAATGTTGTTGATGACAACAGCAAACAAAATGACATGAAGAA

CAAATCGCATATCAATGGCGGTGATGAGTTTGGAAGGTTCTTTTTCAATAGTATTGGCAGT

AACCCATATGAATGCTCTTCGAGTAGCGATAAATTGCCTCGCTTGGAGTCTCGGGAGGAGA

ATGCGTCGGCGTTGTATAACAAAGCAGGGAAGTCCCTTTGGAATGGTGCCCTAAACCTGCC

TGCTAACTAG

**Protein sequence - New *ERF* like genes**

>Acc33766.1 AcERF208

MDLINSSNKVNSPSSSSSTSSSSKSKRKNQQQSQKDLNNSGGLRFIGVRRRPWGRYAAEI

RDPLTKERHWLGTFDTAEEAALAYDRAARSMHMRMQMGDYNSNSMRTTRTNFVYSDMPPG

SSLTSIISPDDEYSQHPLLFLMNSQQNQPPPPPPPSGFFPVPGDEWIQRNTLTDHHNQQQ

QHQQQLCDEQQIITDGFIMHGDVGGGGSTKTNQHYYCNVDEVEAELPPLPPDISNSYDSR

SMCGANVHGSMWNEEDPMSNLLIDNGRFSDQQTTTGYYYHEAETGKSTVGIESYNNVNVG

FLMESASSSELMNPLFRMTQ

>Acc33767.1 AcERF216

MIEDVKTHVIAEGSVSSSPCQPSNAAMPAKFKGVVPQPNGHWGAQIYANHRRIWLGTFKS

EIEAAMVYDSASLKLRTGDNYRNFPWTNVTVHEPKFQSLHSTEAILNMIRDGSYTSKFAD

FLGSQKGEQELSVNLITVHGHGGFSFKLLFQKELTPSDVGKLNRLVVPKKYAVKYFPVVP

ESTEGNTDDGKVDEVHLFFYDRMMRLWKFRYCYWKSSQSFVFTRGWNRFVKEKGLKENDI

IAFYVCEGRERAKEGHSFCLIDVVYPEGSTNSRVDEGTSQNVDMQVDLRLKLGQNLNCWL

VKKDDEGFENSKVFTRLKPTLNGKKSVRLFGVELN

>Acc33768.1 AcERF144

MYEQTVSNTDFALLESIRRHLLEDSDISDFFSATDAPNTLPIYDPSRSFESIVEERVGER

SDHSPPVEWRKYRGVRRRPWGKFAAEIRDPKKRGARIWLGTYETSEDAAVAYDRAAFKIR

GSRAKLNFPHLIGSNKFEPIRVTPKRKKSAAGCSLSPAAPAELDCDTLIGVFDSEMDTDF

YCL

>Acc33769.1 AcERF217

MEEEPSSMIEDVKTRVITEDSVSSSPCQPSNAGMHPIKDNKAMPTKFKGVVPQPNGHWGA

QIYANHQRIWLGTFKSEIEAAMVYDSASLKLRTGDNYRNFPWTNVTVHEPKFQSLHSTEA

ILNMIKDGSYPSKFAVFLGSQKGEQDLGVNLIKGHGHGGFSCKLLFQKELTPSDVGKLNR

LVIPKKYAVKYFPEVPESTEENIDDGKVDEVYLFFYDRIMRLWKFRYCYWKSSQSFVFTR

GWNRFVKEKGLKENDIIAFYVCEGRERAKEGQPFCAIDVVYTGGSRNSRVDEGRSQNVDT

QVDLHLRLGQNLSCWLVKKDDEEFENSKFFTGLKPTPNGKKSVRLFGVELN

>Acc33770.1 ERF95

MENLPQLLSRNTRRRSSRYSTTYLGVRRRPWGRYAAEIRNPLTKERHWLGTFDTAEEAAL

AYDLSSISFSGIERAKTNFHYPFLALPSPSPPPPPPTPSTPDLELEGELGCVEDDESLVI

ASILQSFALQPSNFSFSS

>Acc33771.1 AcERF109 MEGGKKKAQENSGRGGEAKYRGIRRRPWGKYAAEICDPSKNKSRLWLGTFETAEEAARAY

DRAAYSLRGRQAILNFPNEFHCSSPSHGLDGPSCSRVGVESSQEEQVIEFEYLDDKLLEE

LLGTNEDRHANKISKFP

>Acc33772.1 AcERF32

MDYSSGGGGPSGRGGNVAAGDARGSGRHPVYRGVRRRNSGKWVSEIREPRSPNRIWLGTF

PTPEMAAVAYDVAALALRGRDTELNFPNSASSLPVPMSTAPRDIQEAAASAAAAAGAATD

ALAGWTQVHMNENANRSGGGSEFVDEDLIFDMPNVLVNMAEGMLLSPPRLNVAGDHDTAA

EYAGDHNLWKFP

>Acc33773.1 AcERF210

MEEALRMLNDSLIHQPEPDPFLQPLKRVTTAANKRSLRDGGEGGCGTQLRYRGVRRRPWG

RYAAEIRDPQSKERRWLGTFDTAEEAACAYDCAARAMRGVKARTNFAYPTVTTLPPPPSL

TDNFLSRFHCKKPSQTSVRDIPAPTRSFVSSLSSPHGNFPMPTPQRSTSLNTLLLSNLFK

PSLTCEQLPNVSPSTSVCGNAYENYPGSNQIRPETEYHNPGESDDGLDFFRSEPSGSGLL

QEVLNGFYPKPASKGDELSATQNQNCTNEAFNEMMGIESDHFGLHFDGFGSPAVAHSSIS

VPEESMLCEHVFQFHELVSARPFQ

>Acc33774.1 AcERF168 MEGYSSNNYVGDGGTGGGGARSIYRGVRKRKWGKWVSEIREPGKKTRIWLGSFETPEMAA

AAYDAAALHLRGHGARLNFPELAHSLPQPGGPSAEDVRRAAQEAALRFQNPASSFEAGGS

SSNLAPVTIGLSPSQIQAINESPLESPKMWMDPTGALMLEERAFFSNDVEMDEWDEITDD

SLWDP

>Acc33775.1 AcERF148

MQRHSPPKRPEHGGASTSRNPPSRPSRFTREEETSVMVAALSSVISGSTSHNPPPRPSRF

TSEEETCVMVATLSSVISGSTSTQAADLHLLIIPEPDRCQFCKCEDCVGCNFFPPNPDEK

YDNNKNAGISSNSKNDNKKGGKRKKKNKFRGVRQRPWGKWAAEIWNPKRSARVWLGTFET

EEEAARAYDRAAIEFCGTRAKLNFPVPDSSALAQEGQNSELRVERENPSITDMEIGVIRE

EDTETEWINTMMSFDGDCSGSAATGNSHSF

>Fragment1 AcERF149

MRRHSPPKKPAHGGASTSRHPLQPSSLTREEEASVMVAALSAVISGSTSLPEAGPHLLPI

ILEPDTCQFCKIDGCVGCNFFPPSPSDNNNNNNNISISNHASNSSPSHHNKTVGKRRKKN

KYRGVRQRPWGKWGVEIRDPRRAARVWLGTFETEEEGARAYDKAAIEFHGPRAKLNFPFP

ESSAPPEGGQGSEL

>Fragment2 AcERF164

MCGGAILVELIPRTGDERVEKTAKRQRKKLYMRIRQRPWGKWAAEIRDPRKGVRVWIGTF

NTAEEAARAYDREARKIRGNKALRSLLHAFRISC

**New *AP2* genes**

>Acc31415.1 AcAP2L24 [accession-num=7046373]

MEMITLVKSEVSPVRRRLCEMEGDQKQPQQLLVDKSSATNTTVKRSSRFRGVSRWTGRYE

AHLWDKGSWNVITQRKKGKQGAFDEEESAARAYDLAAINHQVTDYEKKIEMMRNVTKEEY

LTSLRRSSGFSRGVSKYRGAARHHHNGRWEARIGRVFGNKYLYLGTYSTQEEAACAYDIA

AIEYRGINADTRLNMESQPVNTLSDQISSQEPEFTFRSSPYTMGNYGNNPRKQEVLETKI

TISPCNRSPSPTALGLLLRSNMFRELVEKNSNVVDDNSKQNDMKNKSHINGGDEFGRFFF

NSIGSNPYECSSSSDKLPRLESREENASALYNKAGKSLWNGALNLPAN
