# Supplementary material for: Copy number variants in kiwifruit ETHYLENE RESPONSE FACTOR/APETALA2 (ERF/AP2)-like genes show divergence in fruit ripening associated cold and ethylene responses in C-REPEAT/DRE BINDING FACTOR-like genes
Source: PLoS One. 2019 May 13;14(5):e0216120. doi: 10.1371/journal.pone.0216120 (PMC6513069; doi:10.1371/journal.pone.0216120)
Supplement: S1 Fig — (PPTX) [file pone.0216120.s006.pptx]

## Slide 1
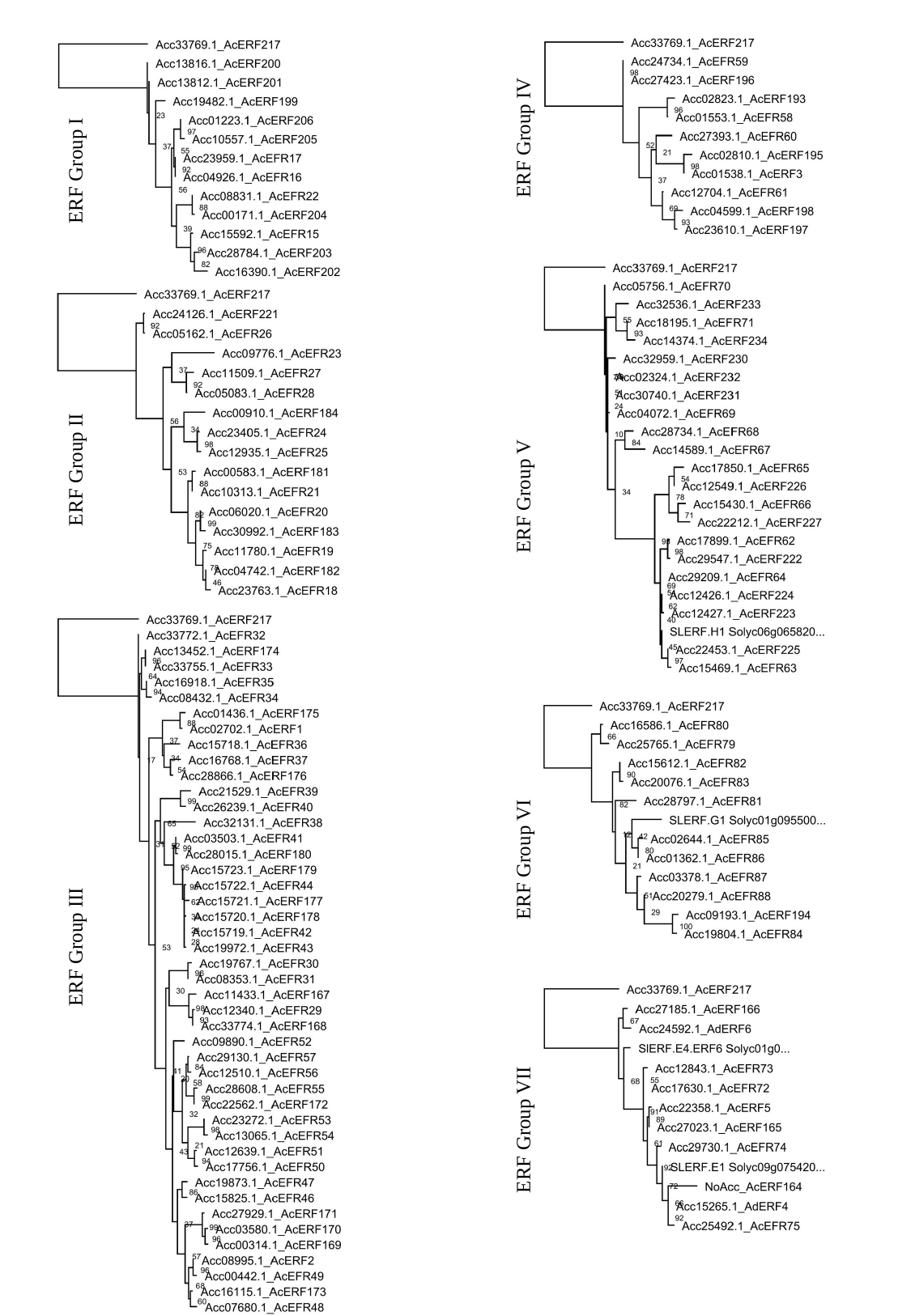

ERF Group IV
ERF Group I
ERF Group II
ERF Group V
ERF Group VI
ERF Group III
ERF Group VII

## Slide 2
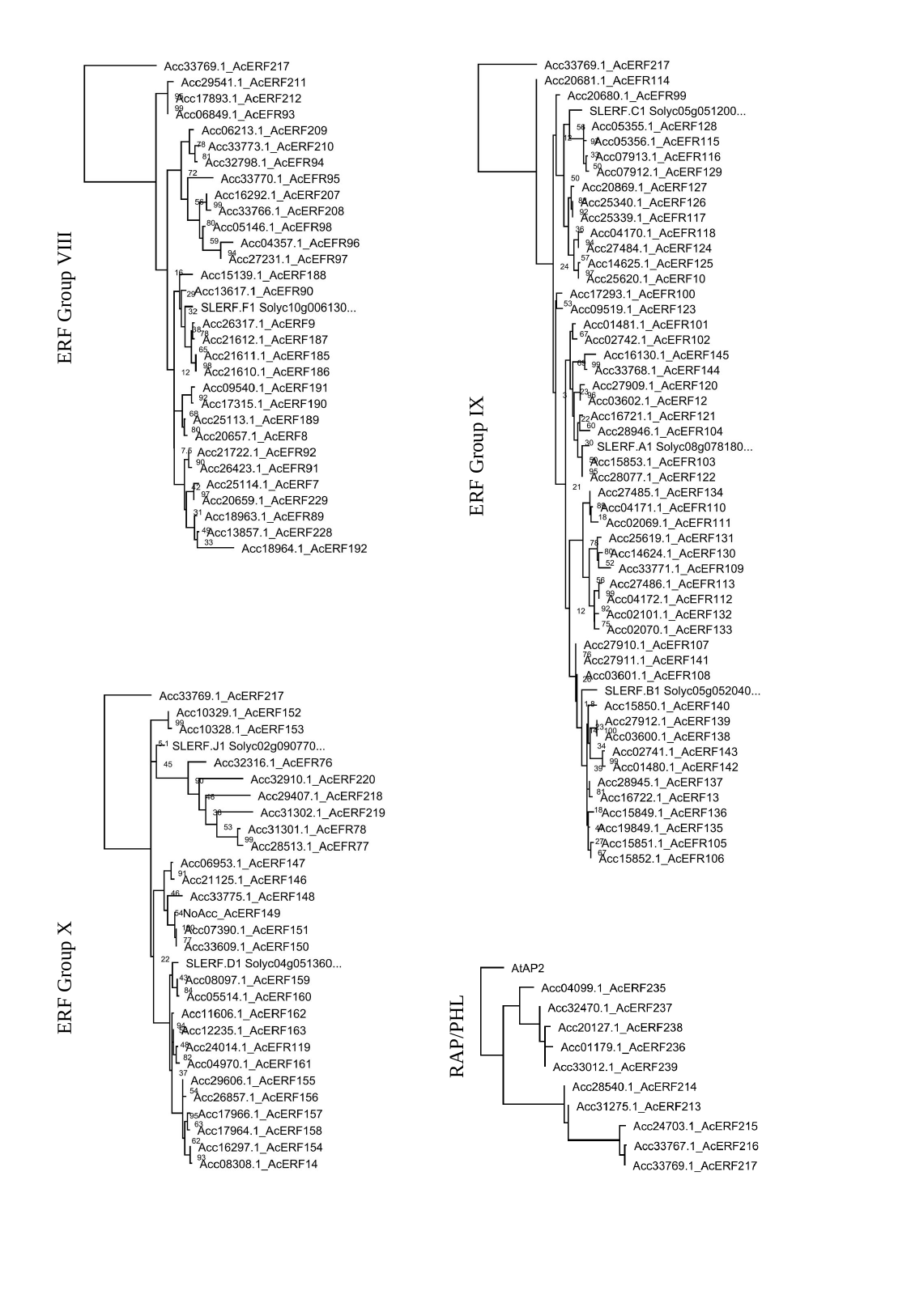

ERF Group VIII
ERF Group IX
ERF Group X
RAP/PHL

## Slide 3
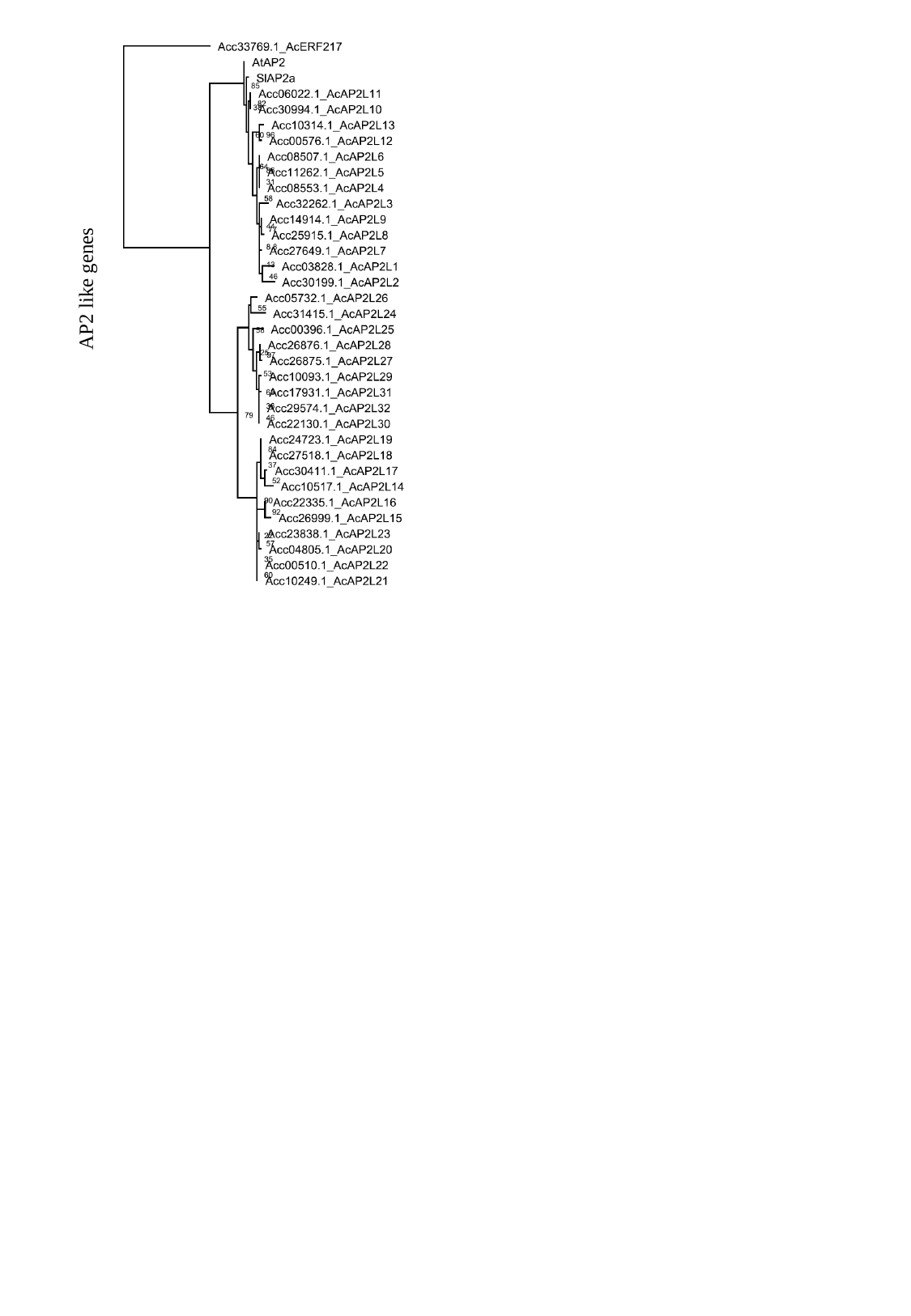

AP2 like genes
